# Flavones Produced by Mulberry Flavone Synthase Type I Constitute a Defense Line against the Ultraviolet-B Stress

**DOI:** 10.3390/plants9020215

**Published:** 2020-02-07

**Authors:** Han Li, Dong Li, Zhen Yang, Qiwei Zeng, Yiwei Luo, Ningjia He

**Affiliations:** 1 State Key Laboratory of Silkworm Genome Biology, Southwest University, Beibei, Chongqing 400715, China; rhanli@163.com (H.L.); lidong870823@swu.edu.cn (D.L.); yangzhen1246305364@163.com (Z.Y.); d2005027@swu.edu.cn (Q.Z.); luoyiwei12@swu.edu.cn (Y.L.); 2 Industrial Engineering Research Center of Mulberry, State Forestry and Grassland Administration, Beibei, Chongqing 400715, China

**Keywords:** flavone synthase, flavone, UV-B radiation, DNA damage, mulberry

## Abstract

Flavones, one of the largest classes of flavonoids in plants, have a variety of bioactivities and participate in the resistance response of plants to biotic and abiotic stresses. However, flavone synthase (FNS), the key enzyme for flavone biosynthesis, has not yet been characterized in mulberry. In this study, we report that the leaves of certain mulberry cultivars, namely BJ7, PS2, and G14, are rich in flavones. We identified a Fe^2+^/2-oxoglutarate-dependent dioxygenase from *Morus notabilis* (MnFNSI) that shows the typical enzymatic activity of a FNSI-type enzyme, and directly converts eriodictyol and naringenin into their corresponding flavones. Overexpression of *MnFNSI* in tobacco increased the flavones contents in leaves and enhanced the tolerance of tobacco to ultraviolet-B (UV-B) stress. We found that mulberry cultivars with higher flavones contents exhibit less UV-B induced damage after a UV-B treatment. Accordingly, our findings demonstrate that MnFNSI, a FNSI-type enzyme, is involved in the biosynthesis of flavones, which provide protection against UV-B radiation. These results lay the foundation for obtaining mulberry germplasm resources that are more tolerant to UV-B stress and richer in their nutritional value.

## 1. Introduction 

Flavones are ubiquitous secondary metabolites in plants and are one of the largest subclasses of flavonoids [1]. Like other flavonoids, flavones have diverse functions that help plants adapt to complex and dynamically changing environments. Flavones play roles in ultraviolet (UV) radiation protection, interspecies interactions, flower coloration, and plant defense [2,3,4,5,6,7,8,9,10]. The continuous thinning of the atmospheric ozone layer in recent years has caused an increase in damage to plants by solar UV radiation. Several studies have shown that the content of flavones is higher in the leaves of plants growing at high altitudes than in those of plants growing at low altitudes, demonstrating a correlation between flavones and plant tolerance to UV stress [11,12]. Other significant properties of certain flavones are their nutritional value and bioactivities in humans, including antioxidant and putative anticancer activity [1,13]. For example, apigenin, a major plant flavone, has antioxidant, anti-inflammatory, and anti-cancer properties, and has potential activity as a chemopreventive and therapeutic agent [14].

The first step in the biosynthesis of flavones is catalyzed by chalcone synthase (CHS), followed by chalcone isomerase (CHI), which produces flavanones as precursors for the subsequent synthesis of flavones and other major flavonoids [15]. Flavone synthase (FNS) is the key enzyme responsible for the conversion of flavanones to flavones, and catalyzes the formation of a double bond between the C2 and C3 of flavanones [1,15]. Two distinct types of FNS enzymes, FNSI and FNSII, can convert the same flavanone substrate to identical products (flavones) by different catalytic mechanisms [1]. The FNSI class belongs to the soluble Fe^2+^/2-oxoglutarate-dependent dioxygenases (2-ODD) superfamily and is mainly found in members of the Apiaceae [1]. The first FNSI enzyme was identified from leaflets of parsley (*Petroselinum crispum*). In enzymatic activity assays, FNSI was able to convert ^14^C-radiolabeled flavanones to the corresponding flavones [16]. Interestingly, flavanone 3β-hydroxylase (F3H) shares about 80% sequence identity with FNSI from the Apiaceae, although they catalyze different reactions in flavonoid biosynthesis [17]. Parsley F3H can be converted to FNSI by site-directed mutagenesis, suggesting that FNSI evolved from F3H in plants [17]. Other FNSIs identified to date include *Arabidopsis thaliana* DOWNY MILDEW RESISTANT6 (AtDMR6), maize ZmFNSI, and rice OsFNSI [18,19]. However, in a phylogenetic analysis, the cluster containing AtDMR6, ZmFNSI, and OsFNSI did not include any FNSI enzymes from the Apiaceae family [18]. In contrast, FNSII enzymes are oxygen and NADPH-dependent cytochrome P450 (CYPs) membrane-bound monooxygenases belonging to the CYP93B and CYP93G subfamilies, which are widespread among higher plants [1].

Mulberry (genus: *Morus*, family: *Moraceae*) is the sole food source of the silkworm and is widely cultivated in China [20]. Several tissues of mulberry (leaves, bark, fruits, and branches) are also used as traditional Chinese medicines because they are rich in a variety of biologically active compounds, such as flavonoids, alkaloids, and polysaccharides [21,22,23,24]. Multiple studies have shown that mulberry flavonoids can prevent headaches and treat hypertension, cardiovascular disease, and diabetes [25,26,27]. Our previous survey of mulberry resources revealed that some mulberry cultivars such as BJ7, PS2, and G14, contain higher total flavonoids contents in their leaves [28]. However, it is unclear why the leaves of these cultivars contain such high levels of flavonoids, or what roles these flavonoids play. In this study, based on metabolomics, in in vitro, and in vivo analyses, we found that flavones are responsible for the differences in flavonoid contents in the leaves among six tested cultivars, including BJ7, PS2, and G14, and identified a FNSI enzyme from *Morus notabilis*. Our results show that MnFNSI is involved in the tolerance of plants to UV-B radiation, and that mulberry cultivars rich in flavones cope with UV-B stress more effectively than do those with lower flavones contents. These results lay the foundation for obtaining mulberry germplasm resources that are more resistant to UV-B stress and richer in nutritional value.

## 2. Results

### 2.1. Differences in Leaf Flavone Contents among Six Mulberry Cultivars

**Figure 1:**
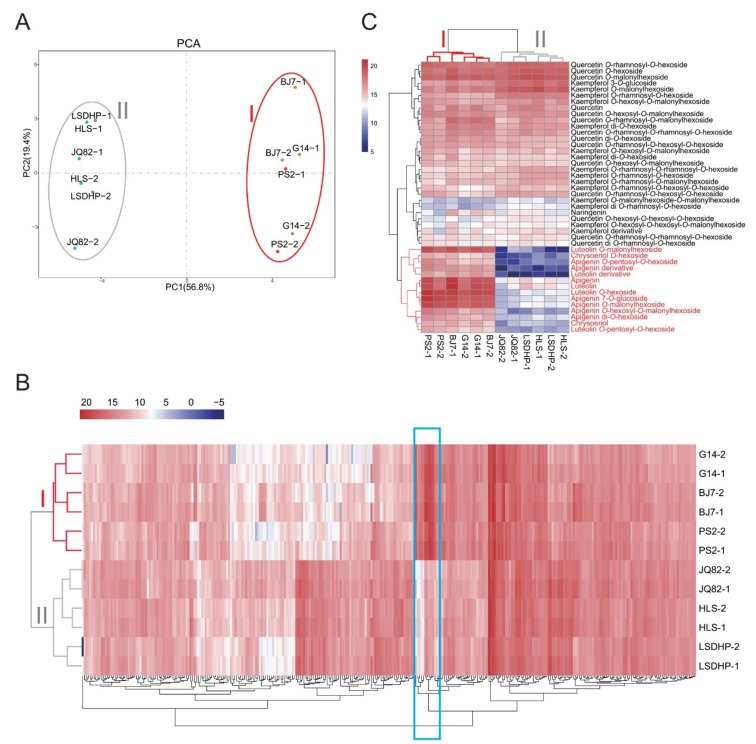
Metabolome analyses of the leaves of six mulberry cultivars. (**A**) PCA score plot of metabolite profiles in leaves of mulberry cultivars BJ7, G14, PS2, HLS, JQ82, and LSDHP. (**B**) Hierarchical clustering of differentially accumulated metabolites in leaves of BJ7, G14, PS2, HLS, JQ82, and LSDHP. Blue box represents flavones. Intensity values were adjusted by log2 transformation, and are represented as colors ranging from white to red. (**C**) Hierarchical clustering of flavonoid metabolites in leaves of BJ7, G14, PS2, HLS, JQ82, and LSDHP. Intensity values were adjusted by log2 transformation, and are represented as colors ranging from navy to red.

Six mulberry cultivars with significant differences in total flavonoids content, namely *Morus alba* L. cv. Baojing 7 (BJ7), *M. alba* L. cv. Pisang 2 (PS2), *M. alba* L. cv. Gui 14 (G14), *M. alba* L. cv. Leshandahongpi (LSDHP), *M. alba* L. cv. Huanglusang (HLS), and *M. alba* L. cv. Jinqiang 82 (JQ82), were selected for liquid chromatography-electrospray ionization-tandem mass spectrometry (LC-ESI-MS/MS) analysis. As shown in Figure 1A, based on a principal component analysis (PCA) of the metabolite profiles, the six cultivars were divided into two groups on the PC1 × PC2 score plot. Group I contained BJ7, PS2, and G14, and group II contained LSDHP, HLS, and JQ82. To identify the differentially accumulated metabolites among these cultivars, orthogonal partial least-squares discriminant analysis (OPLS-DA) was used to model the differences between pairs of mulberry cultivars (Figure S1). In total, 318 compounds were identified as differentially accumulated metabolites (criteria: VIP > 1, *t*-test *p* < 0.05) (Tables S1–S11). The heatmap cluster analysis of these differentially accumulated metabolites indicated that flavones showed the largest differences in concentrations among cultivars, and that the contents of flavones, including apigenin, luteolin and their derivatives, were much higher in BJ7, PS2, and G14 in group I than in LSDHP, HLS, and JQ82 in group II (Figure 1B,C). Flavonols and flavones were main flavonoid compounds in the leaves of these cultivars, but flavonols contents did not differ significantly among the six tested cultivars (Table S2). These results indicate that the significant difference in flavonoid accumulation among the cultivars is caused by the difference in flavones contents, and that flavones strongly contribute to the classification of these cultivars based on their metabolite profiles. 

### 2.2. Putative FNSI Enzyme in Mulberry 

**Figure 2:**
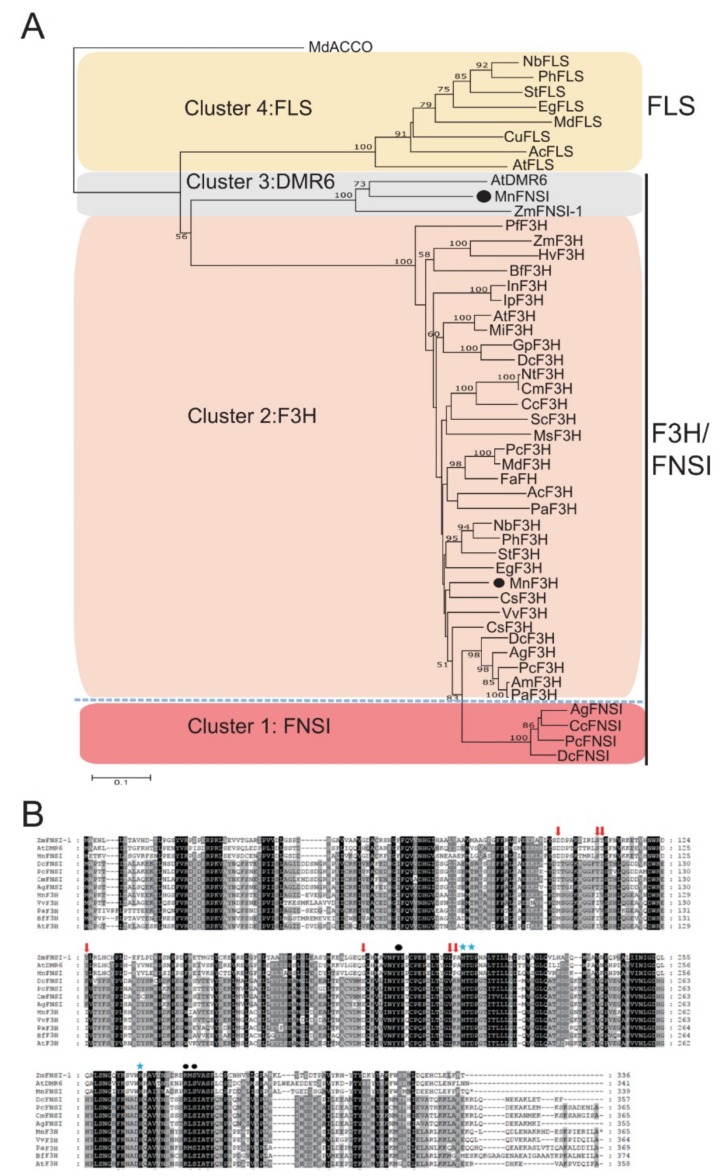
Sequence alignment and phylogenetic analysis of Fe^2+^/2-oxoglutarate-dependent dioxygenases involved in flavonoid biosynthesis. (**A**) Phylogenetic tree constructed with aligned protein sequences with MEGA 5 using the neighbor-joining method with 1000 bootstrap replications. Different Fe^2+^/2-oxoglutarate-dependent dioxygenases are grouped in clusters according to their main demonstrated activities. (**B**) Comparison of MnFNSI proteins with other FNSI and F3H proteins. Red arrow indicates sites determining catalytic activity of FNSI. Asterisks and black dots indicate conserved amino acids necessary for cofactor or substrate binding, respectively.

There are two types of FNS enzymes in plants. Therefore, a BLASTp search of the mulberry protein database (*M. notabilis*) for sequences with the closest homology to other FNSI and FNSII proteins was performed to identify potential FNS candidates. We retrieved two sequences, L484_003477 and L484_028079, with 69% and 49% identity to *Arabidopsis* AtDMR6 and rice CYP93G1, respectively. The leaves of *M. notabilis* are rich in flavones (Table S12), however, we failed to clone *L484_028079*, and the transcripts of *L484_028079* were not detected in the root, branch bark, winter bud, male flower, or leaf of *M. notabilis*. L484_028079 belongs to the CYP93A subfamily, suggesting that it is not a FNSII-type enzyme. In contrast, L484_003477, a typical Fe^2+^/2-oxoglutarate-dependent dioxygenase, was previously identified as a flavanone 3-hydroxylase (MnF3H2) with 36%–38% identity to MnF3H1, apple MdF3H, and petunia PhF3H, and was found to be highly expressed in leaves [29]. We found that L484_003477 showed 34%–36% identity to the FNSI sequences from Apiaceae species, but had high amino acid identity with *Arabidopsis* AtDMR6 and maize ZmFNSI-1 (69% and 63% amino acid identity, respectively). To better classify and identify putative FNS enzymes in mulberry, the amino acid sequence of L484_003477 was used in phylogenetic reconstructions with several other plant 2-ODD proteins that are known to be involved in flavonoid biosynthesis. As shown in Figure 2A, the tree had four clusters corresponding to different enzymatic activities. Enzymes in cluster 1 were FNSIs, those in cluster 2 were F3Hs, and those in cluster 3 and cluster 4 were DMR6 and FLS proteins, respectively. L484_003477 was not in the F3H cluster, but was located in cluster 3. Additionally, sequence alignments of the enzymes from cluster 1, cluster 2, and cluster 3 showed that the site determining FNSI catalytic activity was identical among L484_003477, AtDMR6, and ZmFNSI-1 (Figure 2B). All these findings indicate that mulberry L484_003477 is an FNSI, so it was designated as MnFNSI.

### 2.3. Functional Characterization of Mulberry FNSI

**Figure 3:**
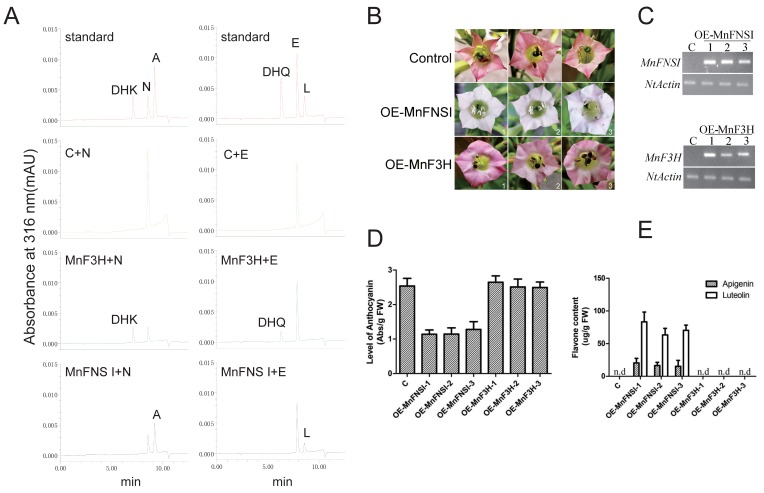
Functional characterization of MnFNSI. (**A**) UPLC chromatograms of products from MnFNSI and MnF3H with naringenin and eriodictyol as substrates. C: bovine serum albumin; A: apigenin; L: luteolin; N: naringenin; E: eriodictyol; DHK: dihydrokaempferol; DHQ: dihydroquercetin. (**B**) Phenotypes of transgenic tobacco harboring *MnFNSI* and *MnF3H*. (**C**) Detection of transgene expression by semi-quantitative RT-PCR analysis. *NtActin* was used as internal control gene. (**D**) Anthocyanin contents in control and transgenic tobacco flowers. (**E**) Flavone contents in flowers of control and transgenic tobacco plants.

To investigate the enzymatic functions of MnFNSI, the coding regions of *MnFNSI* and *MnF3H* were separately expressed in *E. coli* as N-terminal fusion proteins with a His-6 tag. The fusion proteins were purified using Ni^2+^-affinity chromatography, and their enzymatic activities were assayed in vitro (Figure S2 and Figure 3A). When naringenin was used as the substrate in reaction mixtures with MnF3H, the product detected in the UPLC analysis was dihydrokaempferol. In contrast, apigenin was the only product of MnFNSI with naringenin as the substrate. Moreover, MnF3H converted eriodictyol into dihydroquercetin, while MnFNSI converted eriodictyol into luteolin. Transformed tobacco lines overexpressing *MnFNSI* and *MnF3H* were subsequently cultivated to verify the functions of these genes. Compared with the control plants transformed with the empty vector, the plants transformed with *MnF3H* showed no differences in phenotype, while those overexpressing *MnFNSI* had lower anthocyanin contents in flowers (Figure 3B–D). Furthermore, the flavones content was higher in the plants overexpressing *MnFNSI* than in the control plants and those overexpressing *MnF3H* (Figure 3E and Figure S3). These results show that MnFNSI is a flavone synthase, rather than a flavanone 3-hydroxylase.

### 2.4. Regulation of Mulberry FNSI Expression and Flavone Accumulation by UV-B Radiation 

**Figure 4:**
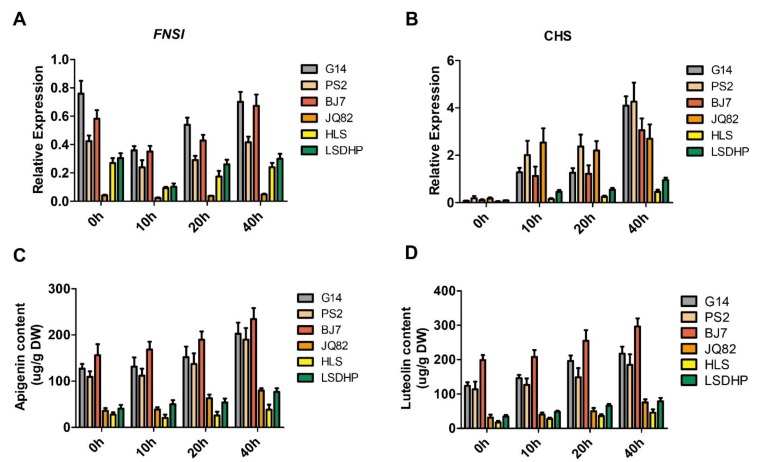
Regulation of *MnFNSI* expression and flavones accumulation by UV-B radiation. (**A**,**B**) Expression analysis of mulberry *FNSI* and *CHS* by qRT-PCR after the UV-B radiation treatment. (**C**,**D**) Variability of flavone contents following UV-B radiation. 0, 10, 20, and 40 h represents 0, 10, 20, and 40 h after the UV-B treatment, respectively.

Next, qRT-PCR analyses showed that the transcript levels of *FNSI* in mulberry leaves were generally higher in the group I cultivars than in the group II cultivars, consistent with the accumulation patterns of flavones in the leaves of the six cultivars (Figure 4A). As flavones may play a role in protecting plants against UV-B radiation, we further examined whether mulberry *FNSI* expression and flavones accumulation in the leaves are regulated by UV-B radiation. After 4 h of UV-B radiation treatment, the flavones contents in the leaves of the cultivars continuously increased, whereas the transcript levels of *FNSI* in these cultivars first decreased and then increased (Figure 4A,C,D). Considering that the biosynthesis of flavones is also controlled by upstream key genes, we performed qRT-PCR analysis to detect *CHS*, the first key gene controlling the flavonoid pathway. As expected, the transcript levels of *CHS* in mulberry leaves increased dramatically upon exposure to UV-B (Figure 4B). These results suggest that the up-regulation of *CHS* results in greater carbon flux into the flavonoid pathway, and that although *FNS* is initially slightly down-regulated under UV-B stress, the ability to biosynthesize flavones is still increased.

### 2.5. MnFNSI Overexpression in Tobacco Decreases UV-B Damage 

**Figure 5:**
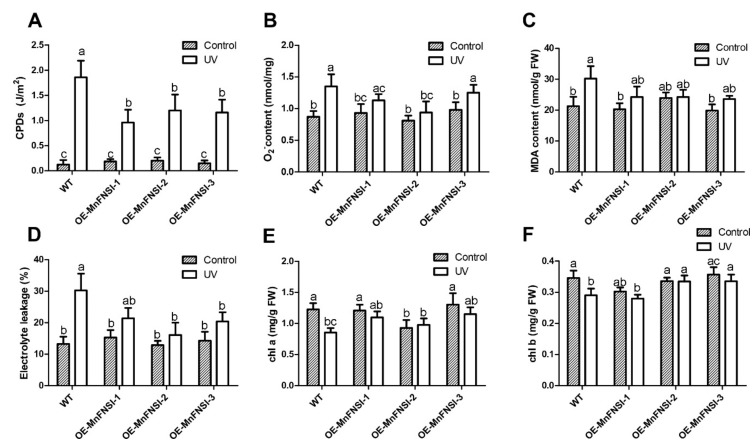
UV-B tolerance analyses of MnFNSI transgenic tobacco plants. Analysis of DNA damage, superoxide accumulation, lipid peroxidation, membrane injury, and chlorophyll content in wild-type and transgenic tobacco overexpressing MnFNI after the UV-B treatment. (**A**) CPDs levels. (**B**) O_2_^−^ content. (**C**) MDA content. (**D**) Electrolyte leakage. (**E**,**F**) chlorophyll content (Chl a and Chl b). Data are means ± SE of three biological replicates from three different experiments. Different lowercase letters indicate significant differences among treatments (One-way ANOVA test, *p* < 0.05).

To investigate whether flavones can protect plants against UV-B damage, wild-type tobacco and transgenic tobacco plants overexpressing *MnFNSI* were cultivated in a growth chamber in the absence of UV-B for 1 month. Plants were then treated with UV-B radiation for 4 h under dark conditions, while control plants were kept in the dark without UV-B treatment. We analyzed DNA damage by measuring the cyclobutane pyrimidine dimers (CPD) contents of these plants. As shown in Figure 5A, wild-type and transgenic tobacco had low levels of CPDs in the absence of UV-B. After UV-B radiation, the CPDs content in all plants was significantly increased, but to a greater extent in wild-type tobacco than in the transgenic lines transformed with *MnFNSI.* This indicated that flavones have a protective effect against DNA damage induced by UV-B. We also analyzed the accumulation of the reactive oxygen species (ROS) by determining the O_2_^−^ contents after UV-B exposure. While the contents of O_2_^−^ increased in all plants after the UV-B treatment, less O_2_^−^ accumulated in the transgenic tobacco than in wild-type tobacco (Figure 5B). We also determined the levels of malondialdehyde (MDA), which is indicative of lipid peroxidation, after the UV-B treatment. Transgenic plants transformed with *MnFNSI* showed a smaller increase in MDA content after the UV-B treatment than did wild-type plants (Figure 5C). The integrity of the cell membranes after UV-B exposure was assessed by determining the leaf electrolyte leakage, which is a measure of oxidative damage. After UV-B radiation, electrolyte leakage increased in all plants, but to a greater extent in wild-type tobacco than in transgenic tobacco (Figure 5D). The chlorophyll (Chl) a and b contents decreased more in wild-type plants than in transgenic plants expressing *MnFNSI* in response to UV-B (Figure 5E,F). These results show that the overexpression of mulberry *FNS* in tobacco plants results in the increased accumulation of flavones, and less UV-B-induced damage.

### 2.6. Mulberry Cultivars Rich in Flavones are Better Able to Cope with UV-B Stress

**Figure 6:**
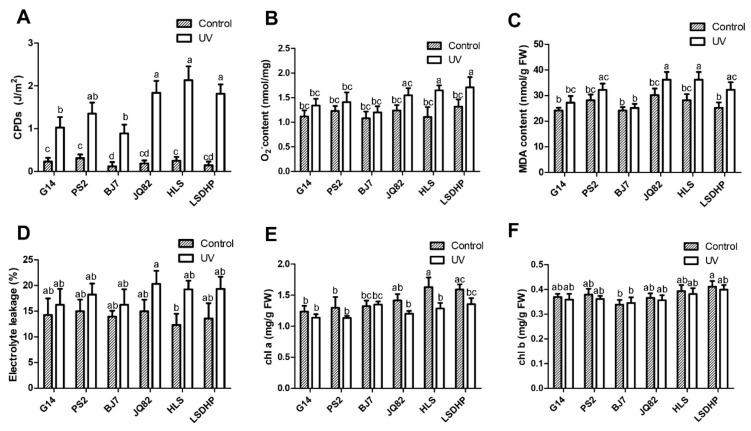
UV-B tolerance analyses of the six mulberry cultivars. Analysis of DNA damage, superoxide accumulation, lipid peroxidation, membrane injury, and chlorophyll content in leaves of mulberry cultivars BJ7, G14, PS2, HLS, JQ82, and LSDHP after the UV-B treatment. (**A**) CPDs levels. (**B**) O_2_^−^ content. (**C**) MDA content. (**D**) Electrolyte leakage. (**E**,**F**) chlorophyll content (Chl a and Chl b). Data are means ± SE of three biological replicates from three different experiments. Different lowercase letters indicate significant differences among treatments (One-way ANOVA test, *p* < 0.05).

To further test whether mulberry cultivars rich in flavones are better equipped to cope with UV-B stress, the six cultivars were cultivated in a growth chamber and then exposed to UV-B radiation for 4 h in the dark. The leaf electrolyte leakage and the contents of CPDs, O_2_^−^, MDA, Chl a, and Chl b were determined. After the UV-B treatment, the CPDs contents in all cultivars were significantly increased, but to higher levels in group II cultivars than in group I cultivars (Figure 6A). Similarly, the contents of O_2_^−^ increased in all cultivars after the UV-B treatment, but to lower levels in G14, PS2, and BJ7 than in the other cultivars (Figure 6B). The MDA content after the UV-B treatment was lower in G14, PS2, and BJ7 than in JQ82, HLS, and LSDHP (Figure 6C). As shown in Figure 6D, in general, the leaf electrolyte leakage increased more in group II cultivars than in group I cultivars after the UV-B treatment. Unlike the Chl a content, the Chl b content in the six cultivars was not significantly affected by UV-B radiation (Figure 6F). The Chl a content in BJ7 was not changed significantly by exposure to UV-B, but in other cultivars it decreased after the UV treatment, and to a greater extent in JQ82, HLS, and LSDHP than in G14 and PS2 (Figure 6E). Thus, mulberry cultivars abundant in flavones are better equipped to cope with UV-B stress.

## 3. Discussion

Flavonoids are the main secondary metabolite in mulberry. Some flavonoids, such as anthocyanin and rutin, have strong antioxidant and anti-inflammatory effects [30,31]. Previous studies have shown that the flavonoid content is higher in mulberry than in other fruits and vegetables, and that the composition and content of flavonoids differ among various mulberry tissues [32]. For example, anthocyanins mainly accumulate in mulberry fruits, while flavonols are the main flavonoid compounds in mulberry leaves [29,31]. In our previous mulberry resource survey, we found that the leaves of some cultivars, including BJ7, PS2, and G14, have high total flavonoid contents [28]. Here, we conducted a metabolomic analysis of the leaves of BJ7, PS2, G14, and another three cultivars, LSDHP, HLS, and JQ82. The cultivars could be divided into two groups based on their metabolite profiles, and flavonoids were the most significant differentially accumulated metabolites. Our results show that the flavonoids in mulberry leaves are mainly flavones and flavonols. There was no difference in flavonol content among the cultivars, but the flavones were found to be much higher in BJ7, PS2, and G14 than in LSDHP, HLS, and JQ82. 

In plants, there are two types of FNS enzymes that control the biosynthesis of flavones: FNSI, which belongs to the 2-ODDs superfamily, and FNSII, which corresponds to a cytochrome P450 monooxygenase [1]. In this study, we identified a 2-ODD protein with homology to AtDMR6 and ZmFNSI (63–69% identity) from *M. notabilis.* This enzyme was named “MnF3H2” in a previous study by Qi et al. [29], but its amino acid sequence is only 36%–38% identical to other F3Hs. Here, in vitro enzyme assays were conducted to identify the function of “MnF3H2”. The results show that “MnF3H2” has typical catalytic activities of a FNSI enzyme, as it can convert naringenin and eriodictyol into apigenin and luteolin, respectively. Additionally, overexpression of “*MnF3H2*” in tobacco reduced the anthocyanin biosynthesis in flowers and increased the flavones content, while the flower color and flavones content of transgenic tobacco overexpressing *MnF3H* did not differ from those of the control plants. All these findings indicate that “MnF3H2” is a FNS, rather than a F3H. Therefore, we renamed it MnFNSI. Our results indicate that higher transcript levels of mulberry *FNSI* in leaves of BJ7, PS2, and G14 result in greater flavone accumulation than in LSDHP, HLS, and JQ82. 

As the ozone layer continues to thin, UV radiation will have more serious effects on plant survival. For example, UV-B radiation can cause cell damage by producing a photoproduct in DNA, and it directly damages proteins and lipids [33]. Flavones have a broad absorption spectrum due to their structural properties, and serve as a shield against the harmful effects of UV-B radiation by accumulating in the outermost cells of epidermal and mesophyll tissue [34]. Previous studies have reported that maysin and rhamnosylisoorientin, two UV-absorbing flavones, accumulate in maize leaves of high-altitude lines in response to UV-B radiation [11]. Overexpression of either the maize FNS enzyme (ZmFNSI and ZmFNSII) in *Arabidopsis* was shown to decrease UV-B induced damage [12]. Additionally, accumulation of apigenin 5-*O*-glucoside in rice enhanced its tolerance to UV radiation [35]. In this study, tobacco transformed with *MnFNSI* accumulated more flavones and exhibited less UV-B induced damage compared with wild-type plants. 

One of the targets of UV radiation is DNA [33]. A major type of DNA damage induced by solar UV radiation is CPDs, which form between adjacent pyrimidine nucleotides on the same DNA strand and inhibit transcription and replication [33,36]. We found that less CPDs accumulated in flavone-rich transgenic tobacco than in wild-type plants after a UV-B radiation treatment, indicating that the accumulation of flavones in tobacco by overexpression of *MnFNSI* reduced DNA damage. Another target is cell membranes, which are subject to lipid peroxidation by ROS induced by UV-B [33]. The ROS react with the side chains of phospholipids containing polyunsaturated fatty acids on cell membranes to form lipid peroxidation products, such as MDA. This alters the fluidity and permeability of cell membranes, leading to changes in cell structure and function [33,37,38]. In addition, MDA may degrade photosynthetic pigments, thereby inhibiting photosynthesis [39]. Here, after a UV-B radiation treatment, the ROS and MDA levels were lower in transgenic lines overexpressing *MnFNSI* than in wild-type tobacco, and the transgenic lines were more resistant to membrane damage and degradation of photosynthetic pigments. Some flavones act as ROS scavengers and sunscreens due to their high UV-B and UV-A absorbing properties [40,41]. Accordingly, based on the results of this study, we proposed that the high levels of flavones in tobacco overexpressing *MnFNSI* absorb UV-B in the outermost cells of epidermal and mesophyll tissue, thereby reducing DNA damage and ROS formation and increasing the tolerance of plants to UV-B stress. 

Flavones usually exist in their decorated forms, which are produced by the activities of glycosyltransferases, acyltransferases, hydroxylases, and methyltransferases [42,43]. Glycosylation of flavones confers structural complexity and different biological activities [35]. It was recently reported that *O*-glycosylated flavones play a positive role in UV-B protection in rice, and flavone 5-*O*-glucosides are more effective UV-B protectants than flavone 7-*O*-glucosides [35]. Our results show that mulberry leaves contain various *C*-glycosyl flavones. Our results also show that, after UV-B irradiation, mulberry cultivars rich in *C*-glycosyl flavones are less susceptible to DNA damage and more resistant to ROS accumulation, lipid peroxidation, membrane damage, and degradation of photosynthetic pigments. Combined with the fact that the transgenic tobacco lines showed an increased tolerance to UV-B radiation, these results suggest that flavones can protect mulberry plants against UV stress. However, which *C*-glycosyl flavones have protective functions in the UV-B tolerance of mulberry plants is unclear. Additional studies are needed to demonstrate the roles of different flavone *O*-glucosides in UV-B protection.

Our results show that overexpression of *MnFNSI* in tobacco increases the flavones content and enhances tolerance to UV-B stress. Our findings also show that mulberry cultivars rich in flavones cope with UV-B stress more effectively than do those with lower flavones contents. While the expression of mulberry *FNSI* differs from that of other *FNS*s, which are up-regulated immediately in response to UV stress, the flavones content in mulberry leaves increased steadily after a UV-B treatment. As the gateway to the entire flavonoid pathway, *CHS* is the first key enzyme controlling the biosynthesis of several downstream pathways, including flavones, flavonols, and anthocyanins [15]. The expression of *CHS* in mulberry leaves was greatly up-regulated after a UV-B treatment, suggesting that there is increased carbon flux into the flavonoid pathway. Therefore, despite the slight decrease in the transcript level of *FNS*, the biosynthesis of flavones will continually increase. Taken together, these results indicate that the flavones produced by MnFNSI participate in defense against UV stress in plants. These results will be useful to identify and breed mulberry germplasm resources that are more tolerant to UV-B stress and richer in nutritional value.

## 4. Materials and Methods

### 4.1. Plant Materials

*M. notabilis* C. K. Schneid, the mulberry species grown in Sichuan province, China, was used to clone *FNSI* and *F3H* genes. Other mulberry cultivars, including *M. alba* L. cv. Baojing 7 (BJ7), *M. alba* L. cv. Pisang 2 (PS2), *M. alba* L. cv. Gui 14 (G14), *M.alba* L. cv. Leshandahongpi (LSDHP), *M. alba* L. cv. Huanglusang (HLS), and *M. alba* L. cv. Jinqiang 82 (JQ82) were grown in a mulberry garden of Southwest University. For UV-B treatments, mulberry branches with leaves were hydroponically cultured in a growth chamber in the absence of UV-B. The branches were then treated with UV-B radiation (power: 20W, wavelength: 311nm, brand: Philips) for 4 h under dark conditions, while control branches were placed in the dark without UV-B radiation. Leaves were harvested at 0, 10, 20, and 40 h. To analyze the tolerance of transgenic tobacco in UV-B stress, wild-type tobacco and transgenic plants were cultured in the chamber for 1 month. Plants were then exposed to UV-B radiation (power: 20W, wavelength: 311nm, brand: Philips) for 4 h under dark conditions, while control plants were placed in the dark without UV-B radiation. Leaves were harvested after UV-B radiation treatments for analysis. 

### 4.2. Chemicals

All solvents and reagents were of analytical grade. Methanol, acetonitrile, phosphoric acid, and acetic acid (HPLC grade) were purchased from ThermoFisher Scientific (Shanghai, China). Authentic standards of apigenin, luteolin, naringenin, eriodictyol, dihydrokaempferol, and dihydroquercetin were purchased from Shanghai Yuanye Biotechnology Co., Ltd. (Shanghai, China). 

### 4.3. Metabolomics Analysis

LC-ESI-MS/MS system (UHPLC, Thermo Scientific™ Dionex™ UltiMate™ 3000; MS, Q Exactive hybrid quadrupole-Orbitrap mass spectrometer; Thermo Fisher Scientific, Waltham, MA, USA) was used for high-throughput metabonomics analysis. Separations were performed on an Aquity UPLC BEH C18 (1.7 μm, 2.1 × 150 mm) column. 0.04% (*v/v*) acetic acid and acetonitrile (containing 0.04% acetic acid) were used as mobile phases A and B, respectively. The gradient conditions were as follows: 95:5 VA/VB at 0 min, 5:95 VA/VB at 20.0 min, 5:95 VA/VB at 22.0 min, 95:5 VA/VB at 22.1 min, 95:5 VA/VB at 26.0 min. The samples were eluted at a flow rate of 0.25 mL/min. For the analytical MS conditions, the parameters of ESI source operation were sheath gas and 35 arbitrary units, the auxiliary gas, sweep gas, spray voltage, capillary temperature and S-lens RF level were 10 arbitrary units, 0 arbitrary units, 3.5 KV, 350 °C, and 50, respectively. For the detailed MS parameters, MS scan range, resolution, microscans, AGC target and Max IT were 100–1000 *m/z*, 70,000, 1, 1 × 10^6^ and 200 ms, respectively. For the data-dependent MS2 (dd-MS2) quantification method, resolution, microscans, AGC target, Max IT, loop count, topN and isolation window were 17,500, 1, 2 × 10^4^, 100 ms, 5, 5, and 1.0 *m/z*, respectively. The parameters of (N)CE/stepped (N)CE were as follows: nce: 15, 30, 60. The parameters of Apex-trigger were 2–6 s. Instrument tuning and mass calibration were carried out by PierceTM LTQ Velos ESI positive ion calibration solution (Pierce, Rockford, IL, USA). In our previous studies, a MS^2^ spectral tag (MS2T) library with 936 metabolite features was constructed for mulberry leaves using a widely targeted metabolomics approach [44,45]. A metabolic profiling analysis of 91 mulberry resources was also conducted in biological duplicate. All qualitative and quantitative data were downloaded from the mulberry metabolome database (MMHub, https://biodb.swu.edu.cn/mmdb/). The data from 12 samples (six cultivars × two biological replicates) were subjected to PCA and OPLS-DA to detect differences in metabolic composition among the six mulberry cultivars. The data were adjusted by log2 transformation and hierarchical clustered using Rpackage pheatmap (https://cran.r-project.org/web/packages/pheatmap/).

In order to visualize the differences between different groups of samples, the unsupervised dimensionality reduction method PCA was applied in all samples using Rpackage models ropls (http://bioconductor.org/packages/release/bioc/html/ropls.html). 

The R package models ropls was used for the OPLS-DA of the comparison groups, and the cross validation was used to verify the OPLS-DA results [46]. The prediction parameters of OPLS-DA model are R2X, R2Y and Q2, where R2X and R2Y, respectively, represent the interpretation rate of the model to X and Y matrix, and Q2 represents the prediction ability of the model. The closer these three indexes are to 1, the more stable and reliable the model is. When Q2 > 0.5, the prediction ability of the model is better, and when Q2 > 0.9, the model is excellent.

### 4.4. Metabolite Identification and Annotation

Metabolite identification/annotation was based on the accurate *m/z*, retention time (RT), and fragmentation patterns. The accuracy of metabolite identification (from high to low) was divided into four levels (from A to D). Level A was the most accurate identification, indicating that those metabolites had the same RT (±0.1 min) and mass spectra as those of authentic standards. Level B indicates that the match rate of those metabolites were greater than 85% when their main fragments were searched against public databases (MassBank, KNApSAcK, HMDB, and METLIN), or showed specific fragmentation patterns. Metabolites only with confident *m/z* (|error| ≤ 10 ppm) by comparison with references or detected in other species were defined as level C and D (relatively low accuracy). The proportions of metabolites in categories C and D were relatively low.

### 4.5. Cloning and Phylogenetic Analysis of FNSI Gene from Morus Notabilis 

The sequences of flavone synthases, which were downloaded from UniProt protein database (http://www.uniprot.org/) [47], were used as retrieval sequences to carry out a BLASTP search in the mulberry protein database (http://morus.swu.edu.cn/morusdb/) [20]. The total RNA of mulberry leaves (*M. notabilis*) was extracted by RNAiso Plus (Takara, Otsu, Japan), and the PrimeScript RT reagent kit (Takara) was used to synthesize the first-strand cDNAs. Genes were amplified using the primers listed in Table S13. The deduced amino acid sequence was alignment and phylogenetic analyzed by MEGA 5.0 program (Neighbor-Joining method with 1000 bootstrap replications).

### 4.6. In Vitro Characterization of Mulberry FNSI Protein 

The full-length coding sequences of *MnFNSI* (Accession numbers: EXB38596.1) and *MnF3H* (Accession numbers: EXC35356.1) were separately recombined into the pET28a (Novagen, Madison, WI, USA) vector. The *Escherichia coli* Rosetta (DE3), which transformed the recombinant plasmids, were cultured in Terrific Broth medium (100 ng/mL kanamycin) at 37 °C to an optical density of 0.3–0.4 at 600 nm, and 1 mM isopropylthiob-galactoside was then added to the cell cultures to induce the expression of recombinant protein at 16 °C for 24 h. High Affinity Ni-NTA Resin (L00250, GenScript, Nanjing, China) was used to purified the fusion proteins. In Vitro enzyme assays of the fusion protein followed the method of Ferreyra et al. [18]. The purified MnFNSI and MnF3H fusion proteins were dissolved in 100 mm phosphate buffer (PH 6.8). 100 μm substrate, 10 mM ascorbic acid, 10 mM a-ketoglutaric acid, and 0.25 mm ferrous sulfate were added to the MnFNSI or MnF3H assay solution for incubation at 28 °C. After 30 min, ethyl acetate was added to terminate the enzyme reaction. The reaction products were extracted by ethyl acetate three times. The extract was dissolved with 200 μL acetonitrile after evaporation.

### 4.7. Plant Transformation

The full-length coding sequence of *MnFNSI* and *MnF3H* were independently recombined into the vector pLGNL under the control of the CaMV35S promoter, and the recombinant plasmids were then transformed into *Agrobacterium tumefaciens* strain GV3101. The transformation and identification of transgenic tobacco were performed as previously described [48]. When detecting transgene expression, *NtActin* was used as internal control gene in semi-quantitative RT-PCR analysis. The primers used in this study are shown in Table S13. 

### 4.8. Quantification of Anthocyanins and Flavones 

The concentration of anthocyanins was calculated using the formula: Q_Anthocyanins_ = (A_530_ − 0.25 × A_657_) × M^−1^ as described by Luo et al. [49]. For flavones quantification, 12 volumes of acid methanol (1% [*v/v*] HCl in methanol) were used to extract the sample powder for 8 h, and then 12 volumes of chloroform and 6 volumes of water were used for the second extraction. Because flavones in mulberry leaves mainly exist in the form of glycoside derivatization, the flavones content in samples was calculated as aglycones by preparing acid-hydrolyzed extracts. The extract was centrifuged at 3000g for 5 min, then the aqueous phase was collected and incubated at 95 °C for 1 h with 2N HCl. 

Flavones quantification was carried out through an Acquity UPLC system (Waters, Milford, MA, USA). The UPLC conditions were as follows: column, Acquity UPLC BEH C18 column (1.7 μm, 2.1 × 100 mm); solvent system, mobile phase A: 40% (*v/v*) acetonitrile, mobile phase B: 0.2% (*v/v*) phosphoric acid; gradient condition, 0–3 min, 20–27% A; 3–6.5 min, 27–84% A; 6.5–7min, 84–20% A; flow rate, 0.17 mL min^−1^; fractions were monitored at 340 nm, and the concentration of the components was determined by the dose-dependent calibration curve of the standard. The UPLC conditions for detecting the products of in vitro enzyme assays were as follows: solvent system, mobile phase A: acetonitrile, mobile phase B: 0.5% (*v/v*) acetic acid; gradient condition, 0–1 min, 30% A; 1–7 min, 30–80% A; 7–7.5 min, 80–100% A; 8 min, 30% A; flow rate, 0.1 mL min^−1^; fractions were monitored at 316 nm. 

### 4.9. Stress Tolerance Analysis of Mulberry and Transgenic Plants 

After UV-B treatments, plant samples were collected to measure the accumulated levels of CPDs, O_2_^−^, MDA, electrolyte leakage, Chl a, and Chl b. For DNA damage analysis, DNA was isolated from the samples and CPDs were analyzed using a commercial ELISA kit (High Sensitivity CPD ELISA kit Ver.2, Cosmo Bio Co., Ltd., Japan) according to the manufacturers’ instructions. The O2^−^ content and MDA content were measured using the Solarbio reagent kit (Cat#BC1290 and Cat#BC0025, Beijing Solarbio Science and Technology Co., Ltd., Beijing, China). For electrolyte leakage analysis, fully expanded green leaves at the same leaf position were harvested and added to 10 mL distilled water in a tube. The samples were shaken for 3 h, and electrolyte leakage from the leaf was measured using a conductivity meter (SX650, Shanghai San-Xin Instrumentation, Shanghai, China). The samples were subsequently boiled for 30 min and the total conductivity was determined. Data are represented as (initial conductivity/total conductivity) × 100. Total chlorophyll (Chl) was measured using standard procedures described by Wintermans and De Mots [50].

### 4.10. Quantitative Real-Time PCR

The treated mulberry leaves were collected for quantitative Real-Time PCR. qRT-PCRs were completed using a StepOnePlus Real-Time PCR System (Applied Biosystems, Foster City, CA, USA). The program conditions and reaction mixture were set as described by Li et al. [51]. The mulberry *ACTIN3* gene was used as an internal control. Primers for qRT-PCR are listed in Table S3.

### 4.11. Statistical Analyses

Tukey’s test (*p* < 0.05) and one-way ANOVA in GraphPad Prism version 4 were used to analyze the data.

## Supplementary Materials

The following are available online at https://www.mdpi.com/2223-7747/9/2/215/s1, Figure S1: OPLS-DA score plots between different mulberry cultivars. Figure S2: Expression of MnFNSI and MnF3H in *Escherichia coli*. Figure S3. Flavone contents in leaves of wild-type and transgenic tobacco plants. Table S1: Qualitative and quantitative results for metabolites in leaves of BJ7, G14, PS2, HLS, JQ82, and LSDHP. Table S2. Quantitative results for flavonoids of BJ7, G14, PS2, HLS, JQ82, and LSDHP leaves. Table S3: Differentially accumulated metabolites between HLS and BJ7. Table S4: Differentially accumulated metabolites between HLS and G14. Table S5: Differentially accumulated metabolites between HLS and PS2. Table S6: Differentially accumulated metabolites between JQ82 and BJ7. Table S7: Differentially accumulated metabolites between JQ82 and G14. Table S8: Differentially accumulated metabolites between JQ82 and PS2. Table S9: Differentially accumulated metabolites between LSDHP and BJ7. Table S10: Differentially accumulated metabolites between LSDHP and G14. Table S11: Differentially accumulated metabolites between LSDHP and PS2. Table S12: Flavonoids content in *M. notabilis* leaves. Table S13. Primers used in this study. Table S14. Accession numbers of sequences used in this work.

## Abbreviations

CHS	Chalcone synthase
CHI	Chalcone isomerase
FNS	Flavone synthase
F3H	Flavanone 3-hydroxylase
AtDMR6	*ARABIDOPSIS thaliana* DOWNY MILDEW RESISTANT6
BJ7	*Morus alba L.* cv. Baojing 7
PS2	*Morus alba L.* cv. Pisang 2
G14	Morus alba L. cv. Gui 14
LSDHP	*Morus alba L.* cv. Leshandahongpi
HLS	*Morus alba L.* cv. Huanglusang
JQ82	*Morus alba L.* cv. Jinqiang 82
PCA	Principal component analysis
OPLS-DA	Orthogonal partial least-squares discriminant analysis
2-ODD	Fe^2+/^2-oxoglutarate-dependent dioxygenases
CPD	Cyclobutane pyrimidine dimer
ROS	Reactive oxygen species
MDA	Malondialdehyde
Chl	Chlorophyll

## References

[1] Martens S., Mithöfer A. (2005). Flavones and flavone synthases. Phytochemistry.

[2] Schmitz-Hoerner R., Weissenbock G. (2003). Contribution of phenolic compounds to the UV-B screening capacity of developing barley primary leaves in relation to DNA damage and repair under elevated UV-B levels. Phytochemistry.

[3] Morimoto S., Tateishi N., Matsuda T., Tanaka H., Taura F., Furuya N., Matsuyama N., Shoyama Y. (1998). Novel hydrogen peroxide metabolism in suspension cells of *Scutellaria baicalensis* Georgi. J. Biol. Chem..

[4] Ahuja I., Kissen R., Bones A.M. (2012). Phytoalexins in defense against pathogens. Trends Plant Sci..

[5] Peters N.K., Frost J.W., Long S.R. (1986). A plant flavone, luteolin, induces expression of *Rhizobium meliloti* nodulation genes. Science.

[6] Wiseman B.R., Snook M.E. (1995). Effect of corn silk age on flavone content and development of corn earworm (Lepidoptera: Noctuidae) larvae. J. Econ. Entomol..

[7] Hooper A.M., Hassanali A., Chamberlain K., Khan Z., Pickett J.A. (2009). New genetic opportunities from legume intercrops for controlling Striga spp. Parasitic weeds. Pest Manag. Sci..

[8] Yoshida K., Mori M., Kondo T. (2009). Blue flower color development by anthocyanins: From chemical structure to cell physiology. Nat. Prod. Rep..

[9] Lan W., Lu F., Regner M., Zhu Y., Rencoret J., Ralph S.A., Zakai U.I., Morreel K., Boerjan W., Ralph J. (2015). Tricin, a flavonoid monomer in monocot lignification. Plant Physiol..

[10] Tanaka Y., Sasaki N., Ohmiya A. (2008). Biosynthesis of plant pigments: Anthocyanins, betalains and carotenoids. Plant J..

[11] Casati P., Walbot V. (2005). Differential accumulation of maysin and rhamnosylisoorientin in leaves of high-altitudes landraces of maize after UV-B exposure. Plant Cell Environ..

[12] Righini S., Rodriguez E.J., Berosich C., Grotewold E., Casati P., Falcone Ferreyra M.L. (2019). Apigenin produced by maize flavone synthase I and II protects plants against UV-B-induced damage. Plant Cell Environ..

[13] Tapas A.R., Sakarkar D.M., Kakde R.B. (2008). Flavonoids as nutraceuticals: A review. Trop. J. Pharm. Res..

[14] Shankar E., Goel A., Gupta K., Gupta S. (2017). Plant flavone apigenin: An emerging anticancer agent. Curr. Pharmacol. Rep..

[15] Falcone Ferreyra M.L., Rius S., Casati P. (2012). Flavonoids: Biosynthesis, biological functions, and biotechnological applications. Front. Plant Sci..

[16] Martens S., Forkmann G., Matern U., Lukačin R. (2001). Cloning of parsley flavone synthase I. Phytochemistry.

[17] Gebhardt Y.H., Witte S., Steuber H., Matern U., Martens S. (2007). Evolution of flavone synthase I from parsley flavanone 3β-hydroxylase by site-directed mutagenesis. Plant Physiol..

[18] Ferreyra M.L.F., Emiliani J., Rodriguez E.J., Campos-Bermudez V.A., Grotewold E., Casati P. (2015). The identification of maize and Arabidopsis type I flavone synthases links flavones with hormones and biotic interactions. Plant Physiol..

[19] Lee Y.J., Kim J.H., Kim B.G., Lim Y., Ahn J.H. (2008). Characterization of flavone synthase I from rice. BMB Rep..

[20] He N., Zhang C., Qi X., Zhao S., Tao Y., Yang G., Tae-Ho L., Wang X., Cai Q., Li D. (2013). Draft genome sequence of the mulberry tree Morus notabilis. Nat. Commun..

[21] Singab A.N., El-Beshbishy H.A., Yonekawa M., Nomura T., Fukai T. (2005). Hypoglycemic effect of Egyptian Morus alba root bark extract: Effect on diabetes and lipid peroxidation of streptozotocin-induced diabetic rats. J. Ethnopharmacol..

[22] Ji T., Li J., Su S.L., Zhu Z.H., Guo S., Qian D.W., Duan J.A. (2016). Identification and determination of the polyhydroxylated alkaloids compounds with α-glucosidase inhibitor activity in mulberry leaves of different origins. Molecules.

[23] Hu T.G., Wen P., Linhardt R.J., Liao S.T., Wu H., Zou Y.X. (2019). Mulberry: A review of bioactive compounds and advanced processing technology. Trends Food Sci. Technol..

[24] Yu W., Chen H., Xiang Z., He N. (2019). Preparation of Polysaccharides from Ramulus mori, and Their Antioxidant, Anti-Inflammatory and Antibacterial Activities. Molecules.

[25] Mahboubi M. (2019). *Morus alba* (mulberry), a natural potent compound in management of obesity. Pharmacol. Res..

[26] Thaipitakwong T., Numhom S., Aramwit P. (2018). Mulberry leaves and their potential effects against cardiometabolic risks: A review of chemical compositions, biological properties and clinical efficacy. Pharm. Biol..

[27] Lim S.H., Choi C.I. (2019). Pharmacological properties of *Morus nigra* L. (black mulberry) as a promising nutraceutical resource. Nutrients.

[28] Li D., Chen G., Ma B., Zhong C., He N. (2020). Metabolic Profiling and Transcriptome Analysis of Mulberry Leaves Provide Insights into Flavonoid Biosynthesis. J. Agric. Food Chem..

[29] Qi X., Shuai Q., Chen H., Fan L., Zeng Q., He N. (2014). Cloning and expression analyses of the anthocyanin biosynthetic genes in mulberry plants. Mol. Genet. Genom..

[30] Shi P.H., Chan Y.C., Liao J.W., Wang M.F., Yen G.C. (2010). Antioxidant and cognitive promotion effects of anthocyanin-rich mulberry (*Morus atropurpurea* L.) on senescence-accelerated mice and prevention of Alzheimer’s disease. J. Nutr. Biochem..

[31] Zhishen J., Mengcheng T., Jianming W. (1999). The determination of flavonoid contents in mulberry and their scavenging effects on superoxide radicals. Food Chem..

[32] Lin J.-Y., Tang C.-Y. (2007). Determination of total phenolic and flavonoid contents in selected fruits and vegetables, as well as their stimulatory effects on mouse splenocyte proliferation. Food Chem..

[33] Nawkar G.M., Maibam P., Park J.H., Sahi V.P., Lee S.Y., Kang C.H. (2013). UV-induced cell death in plants. Int. J. Mol. Sci..

[34] Monici M., Mulinacci N., Baglioni P., Vincieri F.F. (1993). Flavone photoreactivity. UV-induced reactions in organic solvents and micellar systems. J. Photochem. Photobiol. B.

[35] Peng M., Shahzad R., Gul A., Subthain H., Shen S., Lei L., Zheng Z., Zhou J., Lu D., Nishawy E. (2017). Differentially evolved glucosyltransferases determine natural variation of rice flavone accumulation and UV-tolerance. Nat. Commun..

[36] Britt A.B. (1996). DNA damage and repair in plants. Annu. Rev. Plant Physiol. Plant Mol. Biol..

[37] Sanocka D., Kurpisz M. (2004). Reactive oxygen species and sperm cells. Reprod. Biol. Endocrinol..

[38] Gaschler M.M., Stockwell B.R. (2017). Lipid peroxidation in cell death. Biochem. Biophys. Res. Commun..

[39] Sakaki T., Kondo N., Sugahara K. (1983). Breakdown of photosynthetic pigments and lipids in spinach leaves with ozone fumigation: Role of active oxygens. Physiol. Plant..

[40] Csepregi K., Hideg É. (2018). Phenolic compound diversity explored in the context of photo-oxidative stress protection. Phytochem. Anal..

[41] Williams R., Spencer J., Rice-Evans C. (2004). Flavonoids: Antioxidants or signalling molecules?. Free Radicical Biol. Med..

[42] Lepiniec L., Debeaujon I., Routaboul J.M., Baudry A., Pourcel L., Nesi N., Caboche M. (2006). Genetics and biochemistry of seed flavonoids. Annu. Rev. Plant Biol..

[43] Yonekura-Sakakibara K., Saito K. (2014). Function, structure, and evolution of flavonoid glycosyltransferases in plants. Recent Adv. Polyphen. Res..

[44] Chen W., Gong L., Guo Z., Wang W., Zhang H., Liu X., Yu S., Xiong L., Luo J. (2013). A novel integrated method for large-scale detection, identification, and quantification of widely targeted metabolites: Application in the study of rice metabolomics. Mol. Plant.

[45] Wen W., Li D., Li X., Gao Y., Li W., Li H., Liu J., Liu H., Chen W., Luo J. (2014). Metabolome-based genome-wide association study of maize kernel leads to novel biochemical insights. Nat. Commun..

[46] Westerhuis J.A., Hoefsloot H.C., Smit S., Vis D.J., Smilde A.K., van Velzen E.J., van Duijnhoven J.P.M., van Dorsten F.A. (2008). Assessment of PLSDA cross validation. Metabolomics.

[47] UniProt Consortium (2014). Activities at the Universal Protein Resource (UniProt). Nucleic Acids Res..

[48] Liu C., Xu Y., Feng Y., Long D., Cao B., Xiang Z., Zhao A. (2019). Ectopic expression of mulberry G-Proteins alters drought and salt stress tolerance in tobacco. Int. J. Mol. Sci..

[49] Luo P., Ning G., Wang Z., Shen Y., Jin H., Li P., Huang S., Zhao J., Bao M. (2015). Disequilibrium of flavonol synthase and dihydroflavonol-4-reductase expression associated tightly to white vs. red color flower formation in plants. Front. Plant Sci..

[50] Wintermans J.F.G.M., De Mots A.S. (1965). Spectrophotometric characteristics of chlorophylls a and b and their phenophytins in ethanol. Biochim. Biophys. Acta (BBA)-Biophys. Incl. Photosynth..

[51] Li H., Liang J., Chen H., Ding G., Ma B., He N. (2016). Evolutionary and functional analysis of mulberry type III polyketide synthases. BMC Genom..

