# Supplementary material for: Flavones Produced by Mulberry Flavone Synthase Type I Constitute a Defense Line against the Ultraviolet-B Stress
Source: Plants (Basel). 2020 Feb 7;9(2):215. doi: 10.3390/plants9020215 (PMC7076714; doi:10.3390/plants9020215)
Supplement: Supplementary file 1 [file plants-09-00215-s001.zip › plants-705117-supplementary-for conversion/plants-705117-supplementary-for conversion-Supplementary Figures.docx]

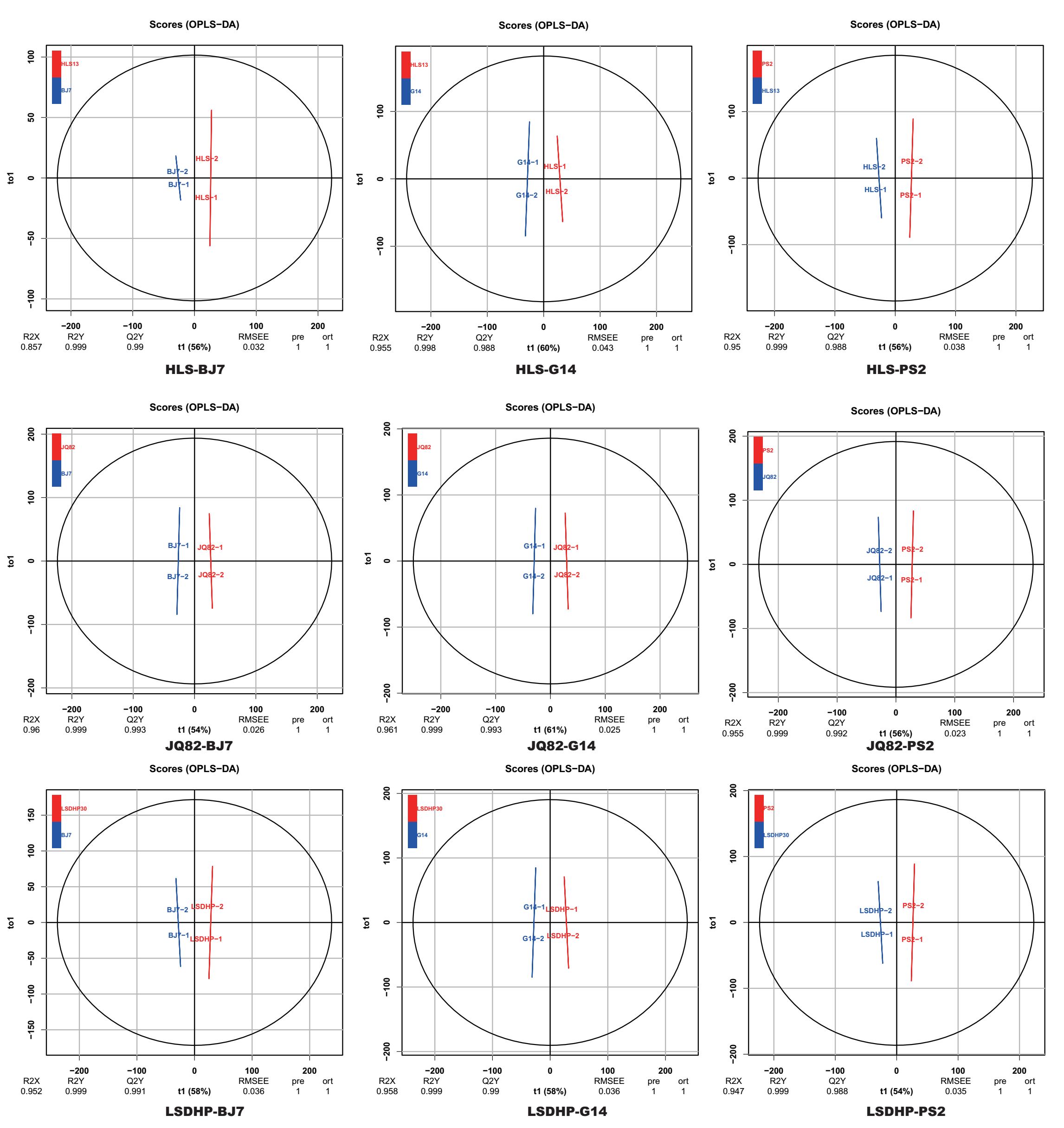


**Figure S1.** OPLS-DA score plots between different mulberry cultivars. Model verification: cross-validation. R2X and R2Y respectively represent interpretation rate of model for X and Y matrices; Q2 represents predictive ability of model. The closer the three indicators are to 1, the more stable and reliable the model is. When Q2>0.5, model prediction ability is good, and Q2>0.9 is an excellent model.


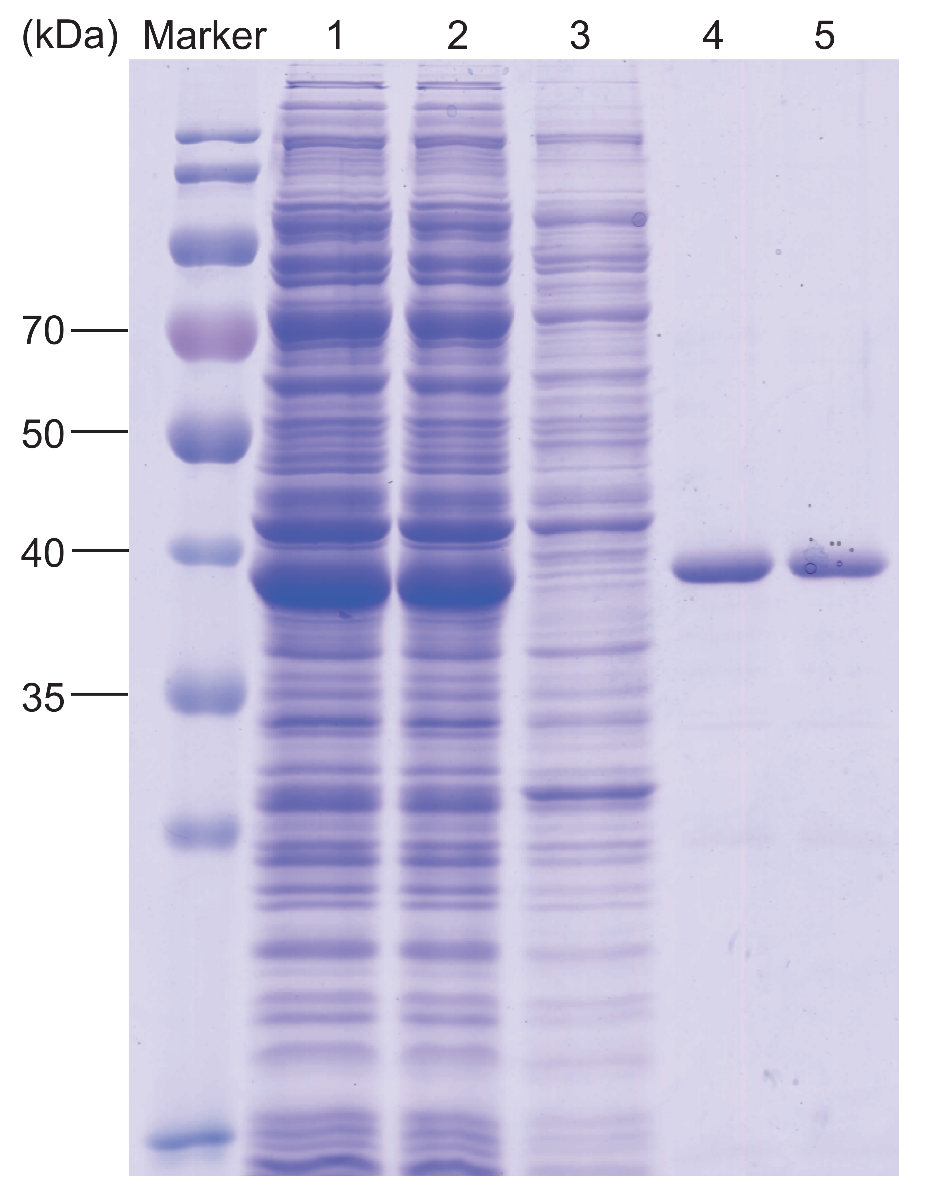


**Figure S2.** Expression of MnFNSI and MnF3H in *Escherichia coli*. Proteins were separated by SDS-PAGE (12%). Lane 1: Soluble protein fraction of pET28a-MnFNSI; Lane 2: Soluble protein fraction of pET28a-MnF3H; Lane 3: Soluble protein fraction of pET28a empty plasmid control; Lane 4: MnFNSI fusion protein purified by Ni2+-affinity chromatography; Lane 5: MnF3H fusion protein purified by Ni2+-affinity chromatography.


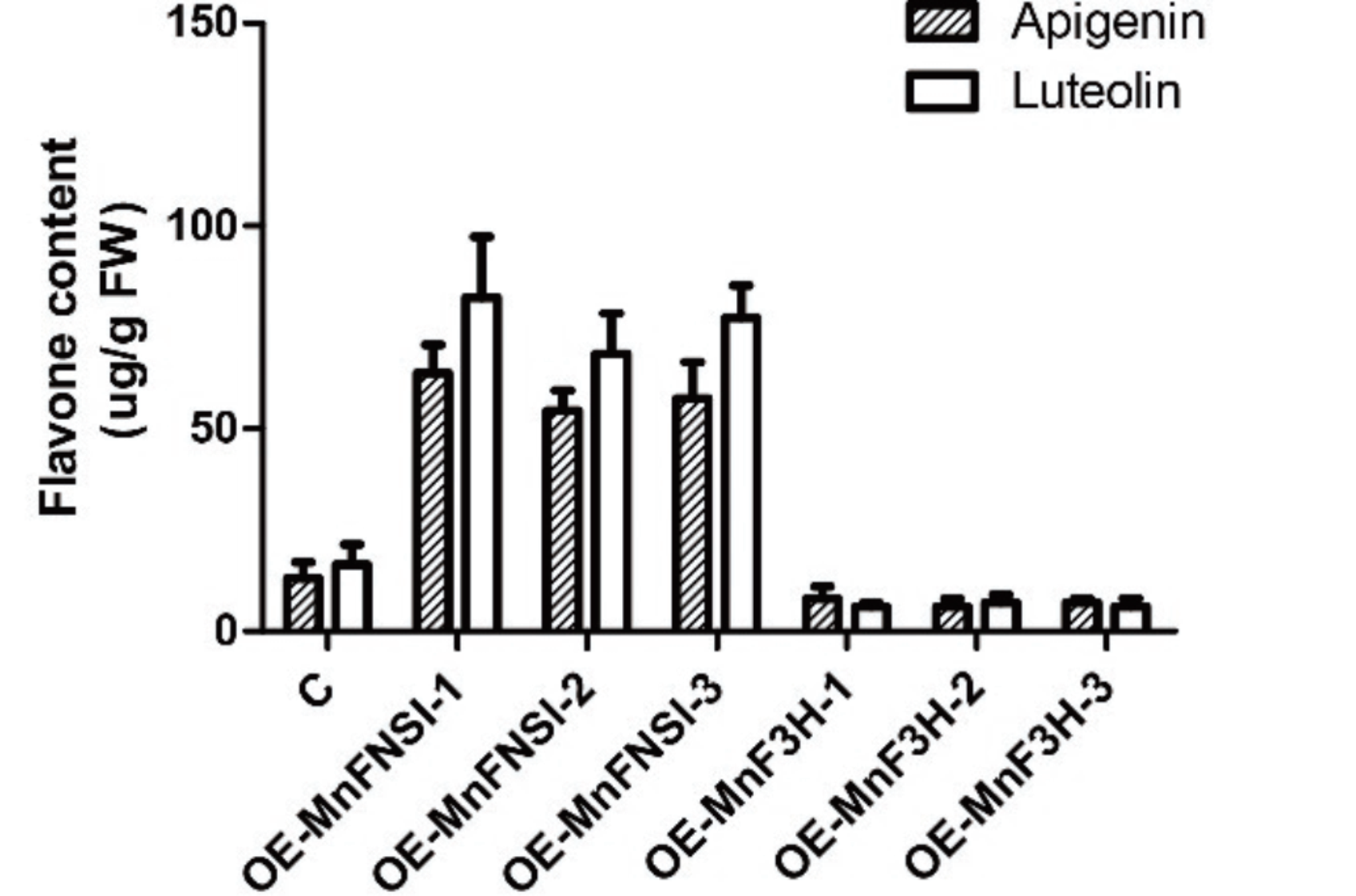


**Figure S3.** Flavone contents in leaves of wild-type and transgenic tobacco plants.
